# Synthesis of Bis(diimino)palladium Nanosheets as Highly Active Electrocatalysts for Hydrogen Evolution

**DOI:** 10.1002/chem.202403082

**Published:** 2024-12-06

**Authors:** Hiroaki Maeda, Eunice Jia Han Phua, Yuta Sudo, Sayoko Nagashima, Wentai Chen, Mayumi Fujino, Kenji Takada, Naoya Fukui, Hiroyasu Masunaga, Sono Sasaki, Kazuhito Tsukagoshi, Hiroshi Nishihara

**Affiliations:** ^1^ Research Institute for Science and Technology Tokyo University of Science 2641 Yamazaki Noda, Chiba 278-8510 Japan; ^2^ Department of Chemistry School of Science The University of Tokyo 7-3-1 Hongo Bunkyo-ku, Tokyo 113-0033 Japan; ^3^ Graduate School of Science and Technology Tokyo University of Science 2641 Yamazaki Noda, Chiba 278-8510 Japan; ^4^ Japan Synchrotron Radiation Research Institute (JASRI) Kouto, Sayo-cho, Sayo-gun, Hyogo 679-5198 Japan; ^5^ Faculty of Fiber Science and Engineering Kyoto Institute of Technology 1 Matsugasaki Hashikami-cho Sakyo-ku, Kyoto 606-8585 Japan; ^6^ RIKEN SPring-8 Center Kouto, Sayo-cho, Sayo-gun, Hyogo 679-5148 Japan; ^7^ Research Center for Materials Nanoarchitectonics (MANA) National Institute for Materials Science (NIMS) 1–1 Namiki Tsukuba 305-0044 Japan

**Keywords:** Bis(diimino)palladium, Coordination nanosheet, Electrochemistry, Electrocatalyst, Heterogeneous catalysis, Hydrogen evolution reaction

## Abstract

Development of efficient electrocatalysts for hydrogen evolution reactions (HERs) is necessary to achieve environmentally friendly and sustainable hydrogen production. To reduce cost and to circumvent the scarcity of platinum, the most efficient catalyst for HER, it is essential to develop catalysts using ubiquitous base metals or minimal amounts of precious metals. Bis(diimino)metal (MDI) coordination nanosheets are potential HER catalysts because their electric conductivities, two‐dimensionality, and porous structures provide large surface areas and efficient mass and electron transfer. In addition, with sparse metal arrangements in their chemical structures, nanosheets can reduce the amount of metal needed. We synthesized bis(diimino)palladium coordination nanosheets (PdDI) as a coordination polymer composed of bis(diimino)palladium, with semiconducting characteristics, using gas‐liquid interfacial synthesis and electrochemical oxidation. These electrochemically synthesized PdDIs exhibit remarkable catalytic performance with overpotential reaching 10 mA cm^−2^ of 34 mV, a Tafel slope of 47 mV dec^−1^, and an exchange current density of 2.1 mA cm^−2^ after appropriate activation. This performance is closely comparable to that of metallic platinum. An *ex‐situ* investigation of the activation process revealed that reduction of the divalent Pd center in bis(diimino)palladium produced a composite of Pd(0) species and PdDI, combining high catalytic activity with smooth electron transfer.

## Graphical abstract

Bis(diimino)palladium (PdDI) coordination nanosheets were synthesized using gas‐liquid interfacial synthesis and electrochemical oxidation. The catalytic activity evaluation for hydrogen evolution reaction revealed the remarkable catalytic performance of activated PdDI which is comparable to that of metallic platinum despite using a tiny number of precious metals.

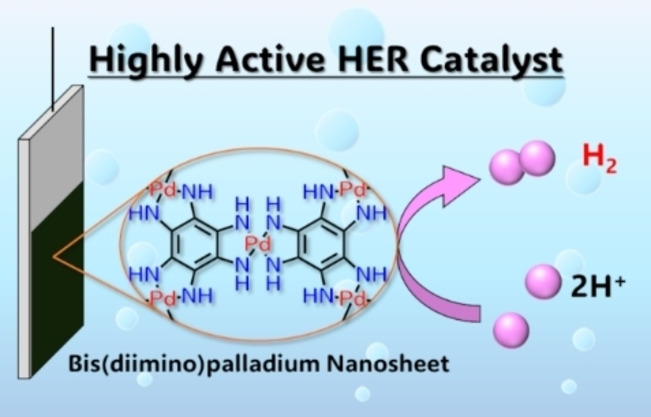

## Introduction

**Figure 1:**
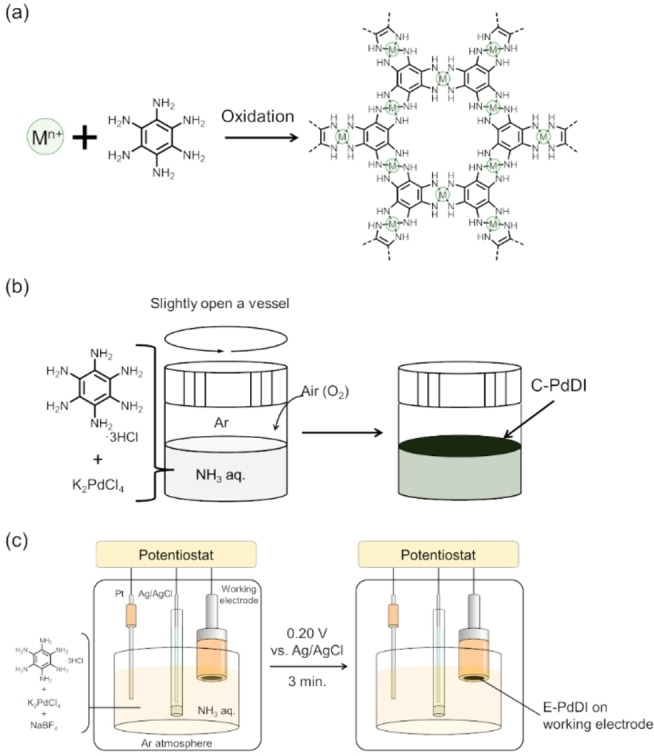
(a) Representative chemical structure of MDI coordination nanosheets composed of metal ions (M^n+^) and hexaaminobenzene (HAB) ligand. Schematic illustration of (b) chemical oxidation at the gas‐liquid interface and (c) electrochemical oxidation methods for PdDI synthesis.

Hydrogen is a clean, efficient energy source for sustainable society because it does not emit CO_2_ and because it can potentially be produced from various sources. It is essential to supply society's energy needs based on hydrogen. However, there are still challenges to achieving mass production, transportation, and storage, and to improving infrastructure.[^1^, ^2^, ^3^, ^4^] Inexpensive, stable hydrogen production is critical to reach the goal. Hydrogen evolution reaction (HER), a cathodic reaction involving electrolysis of water, is considered the most environmentally friendly and sustainable hydrogen production method.[^5^, ^6^, ^7^] Although platinum's catalytic performance is highly efficient, its rarity and cost are problematic. Hence, alternative catalysts have been developed, with[^8^, ^9^, ^10^, ^11^, ^12^, ^13^, ^14^, ^15^, ^16^] or without metals,[^17^, ^18^] or with nano‐amounts of precious metals to reduce their consumption.[^19^, ^20^] π‐Conjugated conductive coordination nanosheets composed of square‐planar metal complexes with four ligating hetero atoms are candidates for efficient HER electrocatalysts because the metal complex sites exhibit HER catalytic activities and their porous, two‐dimensional, π‐conjugated structures provide large surface areas, smooth mass transport, and electron transport between catalytic active sites and electrodes, enhancing catalytic activity.[^21^, ^22^, ^23^, ^24^] Various studies have evaluated HER catalytic performances of coordination nanosheets composed of bis(dithiolato)metals and their analogs.[^25^, ^26^, ^27^, ^28^, ^29^, ^30^] We previously reported electrocatalytic performances of bis(dithiolato)metal complex nanosheets (MDT, M=Pd, Pt)[^28^, ^31^] for HER with overpotentials (*η*_10_) of ca. 410 mV for PtDT and 560 mV for PdDT at a current density of 10 mA cm^−2^.^[28]^ However, further improvement is still required for production of highly active and efficient electrocatalysts. Recently, we directly modified glassy carbon electrodes (GCEs) with bis(diimino)metal coordination nanosheets (MDI, M=Co, Ni, Cu) composed of metal ions and hexaaminobenzene (HAB, Figure 1a) ligands by electrochemical oxidation in the space of a few minutes.[^29^, ^32^, ^33^] NiDI‐modified GCE exhibited the highest HER catalytic performance to date with *η*_10_=227 mV and a Tafel slope of 131 mV dec^−1^.^[32]^ Therefore, we can expect that a PdDI nanosheet composed of palladium ion and HAB ligand will perform as an efficient electrocatalyst. An additional advantage of PdDI nanosheets is their porous framework realizing a low metal atom density, suggesting the possibility of realizing a highly active catalyst with a small number of precious metals.

In this study, we newly synthesized PdDI as one of the MDI families using chemical oxidation and electrochemical oxidation, C‐PdDI, and E‐PdDI, respectively (Figure 1b and c). HER catalytic performance investigations revealed the remarkable catalytic activity of activated E‐PdDI which is comparable to that of Pt electrodes. Hence, we succeeded in producing a highly active HER electrocatalyst with fewer palladium atoms than platinum atoms in metallic Pt because of the sparse arrangement of Pd in the PdDI structure. Our findings showcase that PdDI is a useful coordination polymer to produce efficient HER catalysts with small amounts of noble metal.

## Results and Discussion

**Figure 2:**
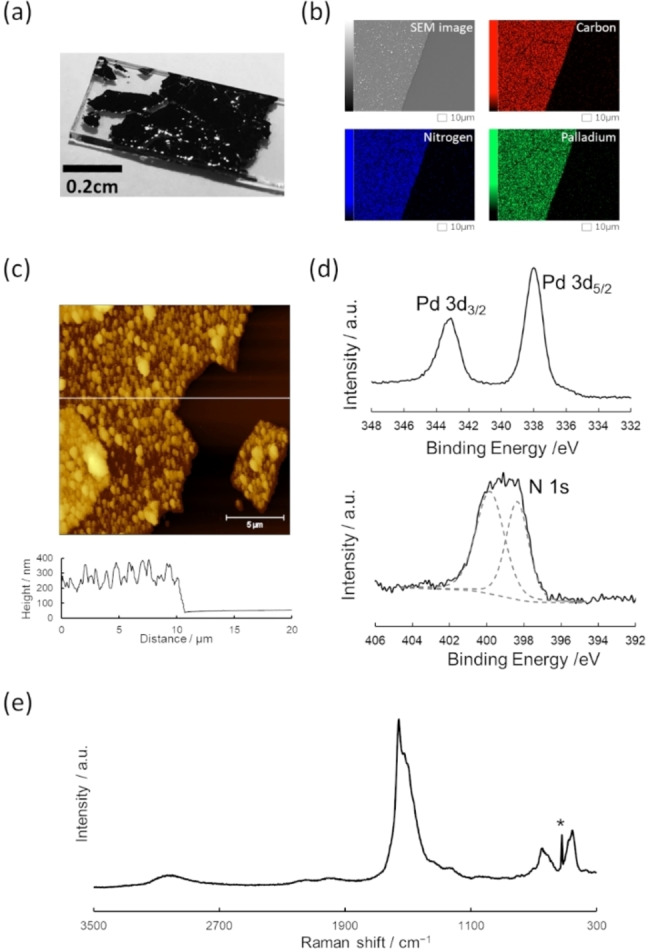
(a) Photograph of C‐PdDI transferred onto a glass substrate. (b) SEM image and EDS mapping, (c) AFM topography image and height profiles at the corresponding white line, (d) XPS of Pd 3d and N 1s regions with the peak deconvolution result (dashed gray lines), and (e) Raman spectrum of C‐PdDI. The peak marked by an asterisk is derived from a Si substrate.

C‐PdDI was synthesized at a gas‐liquid interface by the following procedure. An aqueous ammonia solution of HAB ⋅ 3HCl and potassium tetrachloropalladate(II) (K_2_PdCl_4_) was prepared under Ar. Then, atmospheric oxygen was slowly introduced to the reaction vessel as an oxidizing agent to allow oxidation‐assisted MDI formation (Figure 1b).[^32^, ^34^] The initial colorless solution gradually turned dark yellow, then dark green, and finally PdDI was formed as a black film at the aqueous surface (Figure S1). The resulting PdDI film was transferred onto substrates for characterization (Figure 2a). SEM observations and energy‐dispersed X‐ray spectroscopy (EDS) mapping revealed that C‐PdDI has a sheet‐like structure with a uniform distribution of Pd, N, and C (Figure 2b). AFM topography image revealed a rough surface morphology of C‐PdDI with a sheet‐like structure ca. 150 nm thick. (Figure 2c).

The chemical composition and structure of C‐PdDI were investigated using X‐ray photoelectron spectroscopy (XPS) and Raman spectroscopy. The narrow XP spectra of C‐PdDI on carbon tape showed Pd 3d and N 1s peaks (Figure 2d). The binding energy of Pd 3d peaks at 338.0 and 343.1 eV corresponding to the 3d_5/2_ and 3d_3/2_, respectively, suggested a divalent oxidation state of Pd.[^35^, ^36^, ^37^] The N 1s peak can be deconvoluted into two peaks at 398.4 eV and 399.9 eV, which are assigned to quinoid (C=N) and benzoid (C−N) amines, respectively.[^38^, ^39^, ^40^] The calculated elemental ratio of Pd : N was 1 : 3.8, matching the theoretical ratio (1 : 4) of Pd(*o*‐phenylenediimine)_2_. Raman spectra of C‐PdDI showed a broad peak from 1300 to 1700 cm^−1^ (C=C stretching in an aromatic ring), a peak at 2999 cm^−1^ (N−H stretching), and two peaks at 454 and 644 cm^−1^ corresponding to Pd−N stretching and the breathing mode of the five‐membered PdN_2_C_2_ ring, respectively (Figure 2e).[^41^, ^42^] The IR spectrum also exhibited peaks at 1420 and 3210 cm^−1^ that are assignable to C−N stretching and N−H stretching, respectively (Figure S2). In addition, broad oxidation and reduction waves were observed at ca. 0.23 and ca. 0.0 V vs. ferrocenium/ferrocene (Fc^+^/Fc) in the cyclic voltammogram (Figure S3). These results revealed that C‐PdDI is a coordination polymer consisting of bis(diimino)palladium complexes.

An X‐ray diffraction pattern converted from a grazing‐incidence X‐ray scattering (GIXS) image showed a peak at 2*θ*=17.5°, corresponding to *d*=3.3 Å. This matches typical values of the interlayer distance of MDIs, suggesting that PdDI has a layered structure (Figure S4).[^29^, ^32^, ^33^, ^34^, ^43^, ^44^] Although crystalline MDIs typically give diffraction peaks derived from the in‐plane periodicity in the small 2*θ* range, an observed broad peak around 2*θ*=9° implies low periodicity of C‐PdDI in the in‐plane direction. TEM observations also revealed a film‐shaped structure with an amorphous nature (Figure S5).

Electrical conductivity of pelletized C‐PdDI was 1.3×10^−3^ S cm^−1^ at 300 K using a four‐probe method under helium (Figure S6a). An increase of conductivity with temperature observed in the temperature‐dependent conductivity measurement indicated a semiconductive nature of PdDI with an activation energy of 0.22 eV (Figure S6b).

**Figure 3:**
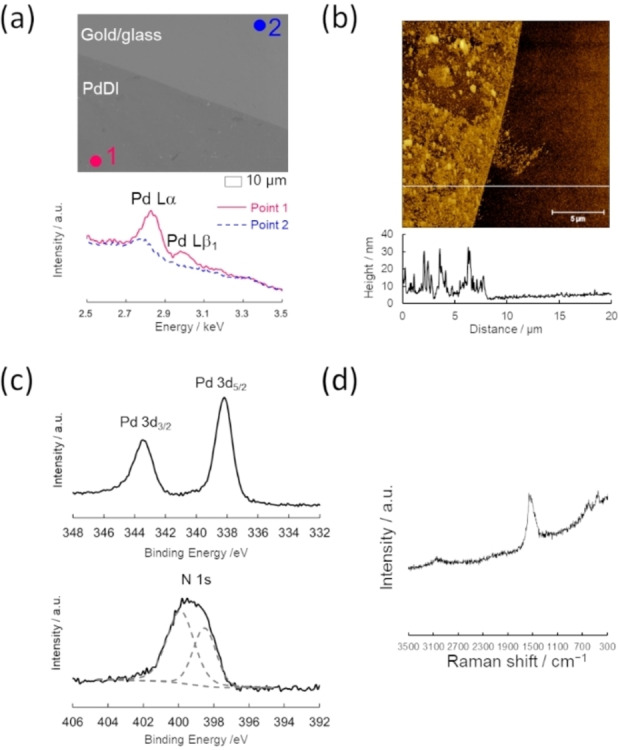
(a) SEM image and EDS spectra recorded at the PdDI‐modified area (point 1: magenta dot) and the bare Au/glass area (point 2: blue dot). (b) AFM topography image and height profiles at the corresponding white line, (c) XPS of Pd 3d and N 1s regions with the peak deconvolution result (dashed gray lines), and (d) Raman spectrum of E‐PdDI formed on an Au/glass electrode.

Synthesis of E‐PdDI was carried out in aqueous ammonia solution in the presence of HAB, K_2_PdCl_4_, and NaBF_4_ by applying an oxidation potential of 0.20 V vs. Ag/AgCl to a working electrode under Ar for 3 min (Figure 1c). Potential for electrochemical oxidation was decided based on the cyclic voltammogram of the precursor solution for the E‐PdDI synthesis (Figure S7). The voltammogram showed redox couples at the potential range between −1 V and −0.4 V and at −0.28 V and an oxidation peak at 0.085 V vs. Ag/AgCl. These peaks can be assigned to the Pd deposition and the first and the second oxidation reactions of the HAB ligand, respectively, from the comparison with the cyclic voltammograms of the HAB ligand and K_2_PdCl_4_ dissolved in 0.1 M NaBF_4_/0.1 M NH_3_ aqueous solutions. Hence, we selected 0.20 V vs. Ag/AgCl as the oxidation potential for E‐PdDI because it is positive enough to oxidize the HAB ligand to promote the PdDI formation. This potential is also positively sufficient to prevent the reduction of Pd(II) ions to Pd(0). Thus, we can eliminate the effect of the palladium deposition on the E‐PdDI synthesis. An SEM image of E‐PdDI formed on an Au/glass electrode shows a clear contrast between PdDI‐modified and bare Au electrode areas. EDS spectra recorded at each area revealed that Pd is present only in PdDI‐modified areas, based upon the peak appearance for Pd Lα (2.83 keV) and Pd Lβ_1_ (2.99 keV) at Point 1, but not at Point 2 (Figure 3a). AFM observation of E‐PdDI prepared on an Au/glass electrode showed a rough film formation 10–30 nm in thickness (Figure 3b). Further characterization of E‐PdDI by XPS and Raman spectroscopy showed good agreement with C‐PdDI described above, indicating a chemical structure of E‐PdDI identical with C‐PdDI (Figure 3c and d). Furthermore, XP spectra of E‐PdDI on an Au/glass electrode recorded at multiple points gave similar peak shapes, positions, and intensities, revealing the uniform formation of E‐PdDI on the electrode surface (Figure S8). Moreover, E‐PdDI exhibited oxidation and reduction peaks at respective potentials of ca. 0.20 and −0.24 V vs. Fc^+^/Fc, corresponding to the redox reaction of [PdN_4_]^+^/[PdN_4_]^0^ (Figure S9).

**Figure 4:**
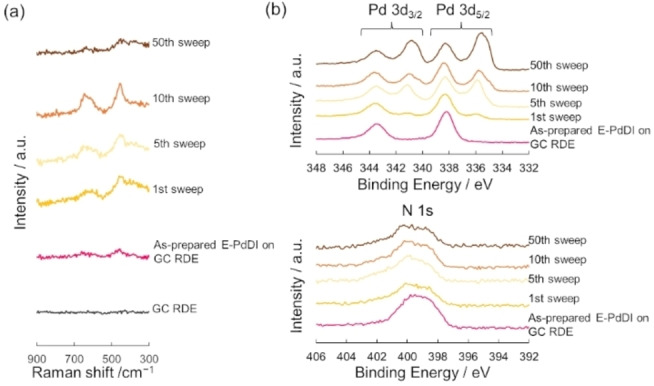
(a) Raman spectra and (b) XPS of E‐PdDI after the 1st, 5th, 10th, and 50th potential sweep in the negative direction.

Linear sweep voltammetry (LSV) of E‐PdDI, C‐PdDI, and E‐NiDI on GC rotating disk electrodes (GC‐RDEs) was performed to evaluate HER catalytic performance in 0.5 M H_2_SO_4_ (pH=0.48) solution at a rotational rate of 1600 rpm. Before electrochemical measurements of the MDIs, cyclic voltammetry (CV) was conducted in the potential range between +0.23 V and +0.93 V vs. RHE for 5 cycles and +0.23 V and −0.37 V vs. RHE for 3 cycles in 0.5 M H_2_SO_4_ solution to remove the residue on the surface. The XP spectra of E‐PdDI after the CV treatment suggested E‐PdDI contains about 15 % of Pd(0) species because applying the negative potential induced the reduction of Pd(II) to Pd(0) (Figures S10, S11). The density of Pd atoms (*N*_Pd_) was calculated as 1.8×10^17^ atom cm^−2^ using the inductively coupled plasma atomic emission spectroscopy (ICP‐AES) (Table S2). As shown in Figure S12, the HER catalytic activity of E‐PdDI was gradually enhanced by repeating potential scans. Raman spectra of E‐PdDI after the 1st, 5th, 10th, and 50th potential sweep in the negative direction did not show a significant difference from the initial spectrum, suggesting that E‐PdDI remained on the electrode (Figures 4a and S13). In contrast, XPS showed the appearance of additional peaks at 335.3 and 340.6 eV assigned to Pd(0) species.^[45]^ The atomic existing ratio of Pd(0):Pd(II):N gradually changed from 0 : 1 : 4.58 in the as‐prepared sample, to 1.55 : 1 : 4.45 with potential sweeps (Figure 4b and Table S1). These results imply that Pd(II) ions in [PdN_4_] complexes were reduced to Pd(0) during the potential sweep in the reductive potential region, resulting in a composite electrocatalyst of Pd(0) species and PdDI. After 50 sweeps, ca. 60 % of Pd(II) in [PdN_4_] was reduced to Pd(0). In addition, the XP spectra recorded at multiple points of E‐PdDI after the 50th potential sweep exhibited that the reduction from Pd(II) to Pd(0) uniformly occurred on the electrode with the ratio of Pd(0) to 69±5 % (Figures S15 and S16). The peak at 399.7 eV in the N 1s region implied the presence of HAB ligand after reducing Pd(II) to Pd(0). ICP‐AES suggested the *N*_Pd_ was maintained at 1.8×10^17^ atom cm^−2^ after 50 sweeps. Furthermore, *η*_10_ values decreased concomitantly with the increase of the Pd(0) atomic ratio (Table S1 and Figure S14). Hence, Pd(0) species potentially act as the most active sites for HER electrocatalysis and the conductive PdDI provides smooth electron transport between the active sites and electrodes.

**Figure 5:**
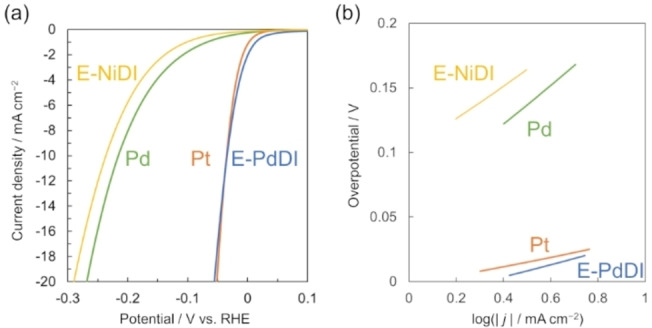
(a) LSV curves and (b) the corresponding Tafel plots of E‐PdDI on GC RDE, E‐NiDI on GC RDE, Pt, and Pd electrodes in a 0.5 M H_2_SO_4_ solution.

Then, we compared the catalytic performance of E‐PdDI activated by multiple LSV scans with the other metal electrodes and MDIs. As shown in Figure 5a, the almost overlapping LSV curves of Pt and newly prepared E‐PdDI after the activation suggest the remarkable HER catalytic activity of E‐PdDI. The E‐PdDI showed an onset potential difference (*E*_onset_) of 13 mV, giving a current density of 1 mA cm^−2^. Furthermore, E‐PdDI exhibited *η*_10_ of only 34 mV, a Tafel slope of 47 mV dec^−1^, and an exchange current density (*j*_0_) of 2.1 mA cm^−2^ (Figure 5b). In addition, the performance of E‐PdDI was greater than C‐PdDI transferred on GC RDE because the directly formed PdDI on the electrodes realizes the smooth electron transport from the electrodes (Table 1, Figure S17). In our previous work, NiDIs prepared using chemical and electrochemical oxidations also exhibited a similar trend.^[29]^ As shown in Table 1, these values are extremely comparable to those of Pt metal electrodes, the most efficient electrocatalysts known for HER. Notably, this performance is significantly superior to that of Pd metal electrodes and reported E‐MDI (M=Ni, Co, Cu) nanosheets,^[29]^ revealing the excellent catalytic activity of E‐PdDI (Figure 5 and Table 1). Considering all Pd(0) species in E‐PdDI contribute to the HER catalysis, the turnover frequency (TOF) of E‐PdDI at *η*_10_=34 mV was estimated as 0.25 s^−1^ based on the *N*_Pd_=1.8×10^17^ atom cm^−2^ from the ICP‐AES and the ratio of Pd(0) from the multiple‐point XPS measurement (69 %) (see details in Supporting Information). However, calculating accurate numbers of active sites in CONASHs is challenging because the active sites on the surface area may prior contribute to catalysis due to the limitation of the accessibility to the active points inside multilayered CONASHs due to their stacking structure.[^46^, ^47^, ^48^] Hence, the actual TOF of E‐PdDI is possibly larger than the estimation in this study. In the case of the Pd metal electrode, only the Pd atoms on the surface can perform as active sites because the electrolyte solution cannot penetrate inside the electrode. The TOF of the Pd electrode at the same potential was calculated as 1.1 s^−1^ based on the *N*_Pd_ on the Pd(111) surface (1.5×10^15^ atom cm^−2^, see details in Supporting Information). In addition, chronopotentiometry measurements at *j*=−10 mA cm^−2^ exhibited that E‐PdDIs maintained the operating potentials for 6 and 12 hours (Figure S18a). The potential fluctuation observed during the measurements may attributed to the oxygen evolution reaction occurring on the counter electrode, which covered the electrode surface with bubbles and inhibited electron transfer. Raman spectra of E‐PdDIs after the 6‐ and 12‐hour measurements showed peaks at 450 and 640 cm^−1^, which were also observed in the as‐prepared E‐PdDI (Figures S18b and c). XP spectra of E‐PdDI after 12‐hour measurements showed that almost all palladium was reduced to zero valence due to the long‐time application of reduction potential. However, the N 1s peak was still observed, suggesting the presence of HAB ligand on the surface (Figure S19). The *N*_Pd_ after the 12‐hour measurement was calculated as 1.6×10^17^ atom cm^−2^ from the ICP‐AES, corresponding to that before the chronopotentiometry experiment (1.8×10^17^). This series of results indicates that the Pd species did not leach even after the reduction from Pd(II) to Pd(0) during the long‐term operation under a highly acidic condition.

**Table 1:** HER electrocatalytic performance.

	*E*_onset_/mV	*η*_10_/mV	Tafel slope/ mV dec^−1^	*j*_0_/mA cm^−2^
E‐PdDI	13	34	47	2.1
C‐PdDI	9	108	99	0.80
Pt	2	35	42	1.2
Pd	−68	216	151	0.40
E‐NiDI	−103	237	127	0.128
E‐CoDI^28)^	−186	340	147	0.048
E‐CuDI^28)^	−386	508	119	0.001

Theoretical calculations by Ji et al. will help understand the superior catalytic activity of E‐PdDI.^[49]^ HER consists of the following three elementary processes.

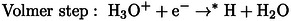




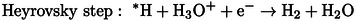




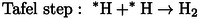




Where *H indicates a hydrogen atom adsorbed on a catalyst. When each process is a rate‐determining step in HER, the Tafel slope ideally takes 120, 40, and 30 mV dec^−1^, respectively.^[50]^ HER can proceed in two pathways, the Volmer‐Heyrovsky and the Volmer‐Tafel processes, and both involve the Volmer step which is the adsorption reaction of a hydrogen atom on a catalyst surface. When the Gibbs free energy of hydrogen adsorption *ΔG*(*H) is near zero, high catalytic performance is expected due to the well‐balanced adsorption and desorption of hydrogen (e. g. *ΔG*(*H) for Pt: −0.09 eV). According to the first‐principles calculations, *ΔG*(*H) of the Pd center in PdDI is 1.45 eV, which is far from zero and close to the *ΔG*(*H) of E‐NiDI as 1.34 eV. Hence, the predicted catalytic performance of PdDI is lower than Pt, but close to NiDI. However, the experimentally observed catalytic performance of activated E‐PdDI is superior to the NiDI. Furthermore, the Tafel slope values of E‐PdDI and NiDI are 47 and 127 mV dec^−1^, respectively, suggesting that their respective rate‐determining steps are the Heyrovsky and Volmer steps. Hence, the difference in the rate‐determining step may contribute to enhancing the catalytic performance of E‐PdDI.

## Conclusions

We created PdDI by chemical and electrochemical oxidation methods. Electrochemically synthesized PdDI on GC electrodes exhibited a catalytic performance with *η*_10_=34 mV, a Tafel slope of 47 mV dec^−1^, and *j*_0_=2.1 mA cm^−2^ after activation. This remarkable performance exceeds that of Pd metal electrodes and conventional MDI (M=Ni, Co, Cu) nanosheets and is extremely comparable to that of Pt electrodes. Summarizing the results, PdDI produces electrocatalysts with remarkable performance despite using very small amounts of precious metals, promising to advance the goal of achieving a hydrogen society.

## Experimental Section

*Materials*: Hexaaminobenzene trihydrochloride (HAB ⋅ 3HCl) was synthesized according to the literature.[^51^, ^52^] Chemical reagents and organic solvents were purchased from commercial sources (TCI, FUJIFILM Wako Pure Chemicals, and ALDRICH) and used without further purification. Commercially available Au/glass (ca. 30 nm gold layer deposited on glass substrates purchased from Kenis) were used as gold substrates. Highly oriented pyrolytic graphite (HOPG) was purchased from Alliance Biosystems, Inc. (Grade SPI‐1/2 10×10×2 mm) and the clean surface was obtained by removing the surface layers with adhesive tape just before use. P‐type Si(100) wafers (≦0.02 Ωcm, AS ONE Corporation) were cut into ca. 1.5×1.5 cm pieces to use as Si substrates.

*Equipment*: Scanning electron microscopy and energy‐dispersed X‐ray spectroscopy were performed using JCM‐7000 NeoScope (JEOL). X‐ray photoelectron spectroscopy data were obtained using PHI 5000 VersaProbe and VersaProbeIII (ULVAC‐PHI). Al Kα (15 kV, 25 W) was used as the X‐ray source, and the beam was focused on a 100 μm^2^ area. The spectra were analyzed using the MultiPak Software and standardized using a C 1s peak at 284.6 eV. Atomic force microscopy (AFM) was carried out using a Hitachi AFM5000II with an SI‐DF40P2 cantilever in the DFM mode. Raman spectra were collected using an NRS‐5500 (JASCO) with a 532‐nm excitation laser. IR spectra were recorded using an FT/IR‐6100 (JASCO) under vacuum conditions. TEM observations were performed at 200 kV of the accelerating voltage using a JEM‐2100F (JEOL). Grading‐incidence X‐ray scattering (GIXS) measurements at λ=1 Å were conducted at Beamline BL05XU in Super Photon ring‐8 GeV (SPring‐8). A HORIBA D‐55S pH meter was used to test the pH values of H_2_SO_4_ solutions for catalytic performance evaluation. Inductively coupled plasma atomic emission spectroscopy was performed using an ICPE‐9820 (Shimazu).

### Synthesis

*C‐PdDI*: 7.5 mL of aqueous potassium tetrachloropalladate (122.4 mg, 375 μmol) solution was prepared with degassed water under an argon atmosphere to obtain a pale‐yellow solution. To this solution, 7.5 mL of concentrated aqueous ammonia was added. 15 mL of the resulting colorless solution was added to 10 mL of 1 mM of aqueous ligand solution and then exposed to atmospheric air at room temperature for over 8 days. A black film was formed at the water surface and black precipitation was formed in the reaction solution.

*E‐PdDI*: Before an electrochemical synthesis, the glassy carbon electrodes were polished with 0.03 μm Al_2_O_3_ powder dispersion on polishing pads, then rinsed with water and dried under argon flow. The polished glassy carbon electrodes for the electrochemical synthesis were pretreated by scanning cyclic voltammetry in 0.1 M H_2_SO_4_ from 0.0 V to 2.2 V (vs. Ag/AgCl) for 25 cycles with a scan rate of 0.1 V/s to activate the surface by anodic polarization. HAB ⋅ 3HCl (4.4 mg, 16 μmol), K_2_PdCl_4_ (7.8 mg, 24 μmol), and NaBF_4_ (0.22 g, 2.0 mmol) were dissolved in a 0.1 M ammonia solution (20 mL) in an Ar‐purged glove box. Electrochemical synthesis was conducted using the glassy carbon as a working electrode (3 mm*φ* or 4 mm*φ*), a Pt coil as a counter electrode, and an Ag/AgCl reference electrode. A constant potential (0.20 V vs. Ag/AgCl) was applied for 3 min to form E‐PdDI on the working electrode. The modified electrode was rinsed with water and then dried under vacuum. Au glass electrodes were also used as working electrodes for electrochemical synthesis.

*E‐NiDI*: E‐NiDI was synthesized according to the preparation procedure for E‐PdDI. For electrochemical oxidation synthesis of E‐NiDI, HAB ⋅ 3HCl (2.2 mg, 8 μmol), Ni(OAc)_2_ ⋅ 4H_2_O (3.0 mg, 12 μmol), and NaBF_4_ (0.11 g, 1.0 mmol) were dissolved in a 0.1 M ammonia solution (10 mL) in an Ar‐purged glove box, and the oxidation potential of 0.58 V vs. Ag/AgCl was applied to the working electrodes.

*Conductivity measurements*: The C‐PdDI obtained from vacuum filtration were first ground using a mortar and pestle before pressing into a pellet. The pelletized form was then approximately sliced into flat rectangular strips for the resistivity measurements. The direct‐current resistivity measurements were performed with the pelletized C‐PdDI using the standard four‐probe method. Electrical contacts were obtained by gluing four gold wires (15 μm diameter) to the pellet with carbon paste.

### Electrochemical Measurements

*Cyclic voltammetry of C‐PdDI and E‐PdDI*: Cyclic voltammetry used a three‐electrode configuration electrochemical cell with 1 M acetonitrile solution of tetrabutylammonium hexafluorophosphate (^*n*^Bu_4_NPF_6_) as the electrolyte solution, a Pt coil as the counter electrode and an Ag^+^/Ag electrode as the reference electrode (an Ag wire immersed in a 10 mM AgClO_4_/ 100 mM ^*n*^Bu_4_NClO_4_/acetonitrile solution). A C‐PdDI/HOPG substrate or an E‐PdDI‐modified GC electrode was used as the working electrode. A 650DT electrochemical analyzer (BAS) performed the potential control and data collection. The recorded potentials were adjusted from Ag^+^/Ag to Fc^+^/Fc using the difference in potentials between the two redox couples.

*Linear sweep voltammetry of E‐PdDI, E‐NiDI, and C‐PdDI for electrocatalytic performance evaluation*: The electrocatalytic performance evaluations were carried out by ALS 750E electrochemical analyzer (BAS) and RRDE‐3A rotating ring disk electrode apparatus (BAS) in a conventional three‐electrode cell. All the electrodes except an Ag/AgCl electrode were purchased from BAS. The Ag/AgCl electrode (an Ag wire in a saturated KCl solution) or reversible hydrogen electrode (RHE) was used as a reference electrode, and a Pt wire was used as a counter electrode. The following equation calibrated the potentials recorded using the Ag/AgCl reference (*E*_Ag/AgCl_) to the potential vs. RHE (*E*_RHE_).
(1)

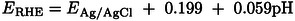




GC RDEs modified with MDIs were used as working electrodes. The E‐PdDI‐ and E‐NiDI‐modified electrodes were prepared using the synthesis method described above. The C‐PdDI‐modified electrodes were prepared by transferring C‐PdDI film synthesized by the gas‐liquid interfacial method using the Langmuir‐Schaefer method. Before the measurements, the solution was fully purged with argon for 30 minutes. Then, cyclic voltammetry (CV) was performed in the potential range between +0.23 V and +0.93 V vs. RHE for 5 cycles and +0.23 V and −0.37 V vs. RHE for 3 cycles in 0.5 M H_2_SO_4_ solution to remove the residue on the surface. The rotation rate was set at 1600 rpm during linear sweep voltammetry measurements.

*Chronopotentiometry measurement of E‐PdDI for long‐term stability test*: Chronopotentiometry was carried out by an HZ‐Pro S4 (Hokuto Denko) using an Ag/AgCl, an activated E‐PdDI on GC RDE, and a Pt wire as a reference, working, and counter electrodes, respectively, in an Ar‐purged 0.5 M H_2_SO_4_ solution. The rotational rate was set at 1600 rpm during the measurements.

*Inductively coupled plasma atomic emission spectroscopy*: Replaceable GC RDEs (4 mm*φ*) were used as working electrodes for E‐PdDI synthesis. After the CV treatment, 50‐cycle LSV measurement, and 12‐hour chronopotentiometry measurement, the GC disks were detached from the electrode attachments and immersed into HCl/H_2_O_2_ solution (1 mL: 1 mL) to decompose and dissolve E‐PdDIs on the electrodes. The solutions were diluted to 50 mL for the measurements. The number of Pd atoms in the unit area (*N*_Pd_ atom cm^−2^) was calculated from the following equation:

(2) $$N_{Pd}=\frac{c\times 10^{-6}\times 50{\times N}_{A}}{M_{Pd}\times 1000\times \left(0.2\times 0.2\times \pi \right)}$$

Where, *c*, *N*_A_, and *M*_Pd_ indicate the concentration of Pd detected by ICP measurement (ppb), the Avogadro constant (6.02×10^23^ atom mol^−1^), and the molar mass of Pd (106.42 g mol^−1^).

## Conflict of Interests

The authors declare no conflict of interest.

1

## Supporting information

As a service to our authors and readers, this journal provides supporting information supplied by the authors. Such materials are peer reviewed and may be re‐organized for online delivery, but are not copy‐edited or typeset. Technical support issues arising from supporting information (other than missing files) should be addressed to the authors.

Supporting Information

## Data Availability

The data that support the findings of this study are available from the corresponding author upon reasonable request.
