# Supplementary material for: Synthesis of Bis(diimino)palladium Nanosheets as Highly Active Electrocatalysts for Hydrogen Evolution
Source: Chemistry. 2024 Dec 6;31(6):e202403082. doi: 10.1002/chem.202403082 (PMC11771586; doi:10.1002/chem.202403082)
Supplement: Supplementary file 1 — Supporting Information [file CHEM-31-e202403082-s001.pdf]

Chemistry–A European Journal

Supporting Information

Synthesis of Bis(diimino)palladium Nanosheets as Highly Active Electrocatalysts for Hydrogen Evolution

Hiroaki Maeda,* Eunice Jia Han Phua, Yuta Sudo, Sayoko Nagashima, Wentai Chen, Mayumi Fujino, Kenji Takada, Naoya Fukui, Hiroyasu Masunaga, Sono Sasaki, Kazuhito Tsukagoshi, and Hiroshi Nishihara*

Synthesis of Bis(diimino)palladium Nanosheets as Highly Active Electrocatalysts for Hydrogen Evolution

Hiroaki Maeda,*[a] Eunice Jia Han Phua,[b] Yuta Sudo,[c] Sayoko Nagashima,[a] Wentai Chen,[c] Mayumi Fujino,[c] Kenji Takada,[a] Naoya Fukui,[a] Hiroyasu Masunaga,[d] Sono Sasaki,[e, f] Kazuhito Tsukagoshi,[g] and Hiroshi Nishihara*[a, c]

[a] Dr. H. Maeda, Dr. S. Nagashima, Dr. K. Takada, Dr. N. Fukui, Prof. Dr. H. Nishihara
Research Institute for Science and Technology
Tokyo University of Science
2641 Yamazaki, Noda, Chiba, 278-8510, Japan
E-mail: h-maeda@rs.tus.ac.jp; nisihara@rs.tus.ac.jp

[b] Dr. E. J. H. Phua
Department of Chemistry, School of Science
The University of Tokyo
7-3-1 Hongo, Bunkyo-ku, Tokyo 113-0033, Japan

[c] Y. Sudo, W. Chen, M. Fujino, Prof. Dr. H. Nishihara
Graduate School of Science and Technology
Tokyo University of Science
2641 Yamazaki, Noda, Chiba, 278-8510, Japan

[d] Dr. H. Masunaga
Japan Synchrotron Radiation Research Institute (JASRI)
Kouto, Sayo-cho, Sayo-gun, Hyogo 679-5198, Japan

[e] Prof. Dr. S. Sasaki
Faculty of Fiber Science and Engineering
Kyoto Institute of Technology
1 Matsugasaki Hashikami-cho, Sakyo-ku, Kyoto 606-8585, Japan

[f] Prof. Dr. S. Sasaki
RIKEN SPring-8 Center
Kouto, Sayo-cho, Sayo-gun, Hyogo, 679-5148, Japan

[g] Prof. Dr. K. Tsukagoshi
Research Center for Materials Nanoarchitectonics (MANA)
National Institute for Materials Science (NIMS)
Namiki 1-1, Tsukuba 305-0044, Japan

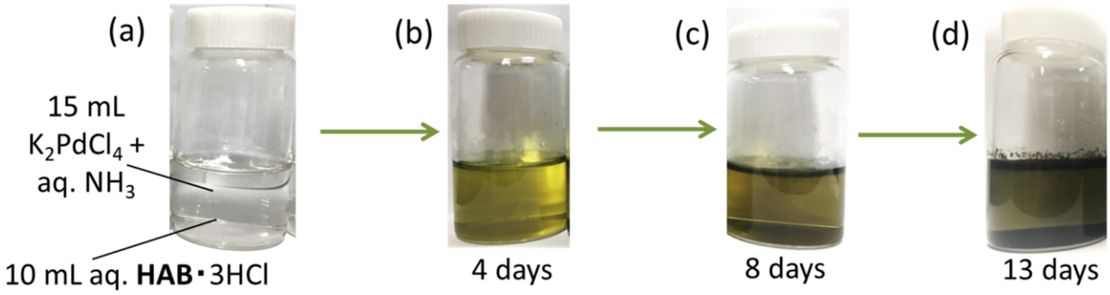

Fig. S1. Photographs taken during the formation of C-PdDI. (a)Beginning of experiment (0 hours). (b)After 4 days. (c)After 8 days. (d)After 13 days.

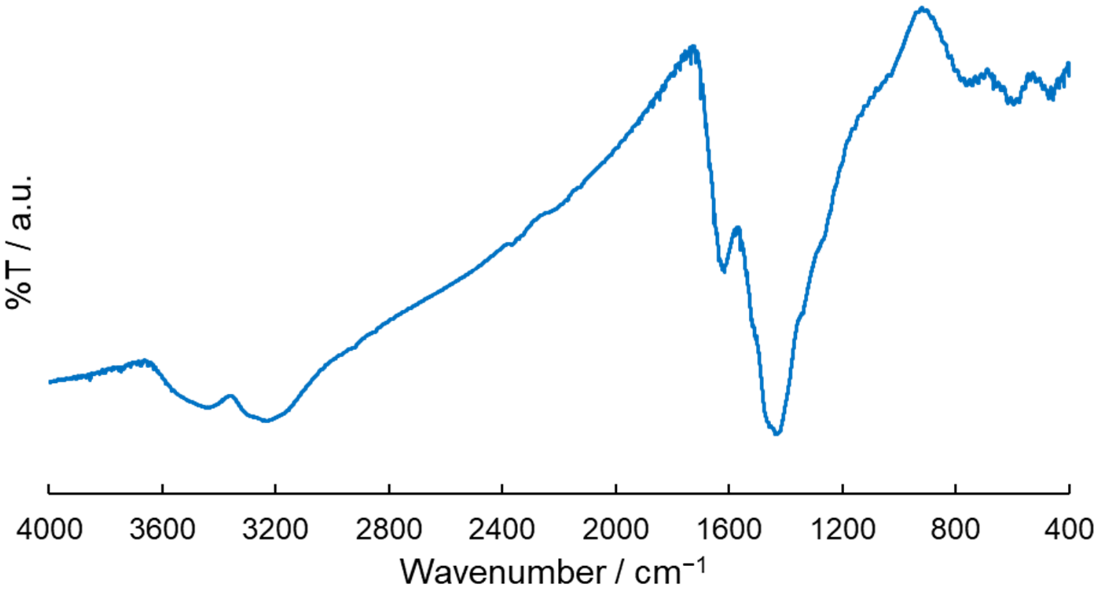

Fig. S2. IR spectrum of C-PdDI.

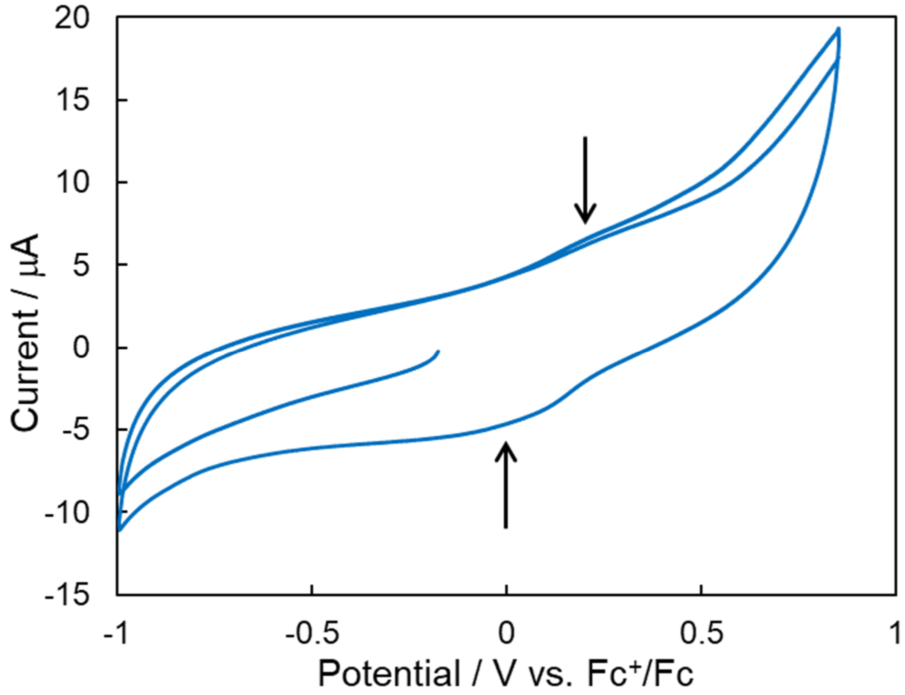

Fig. S3. Cyclic voltammogram of C-PdDI on a HOPG in 1 M $^{n}Bu_4NClO_4/CH_3CN$ solution.

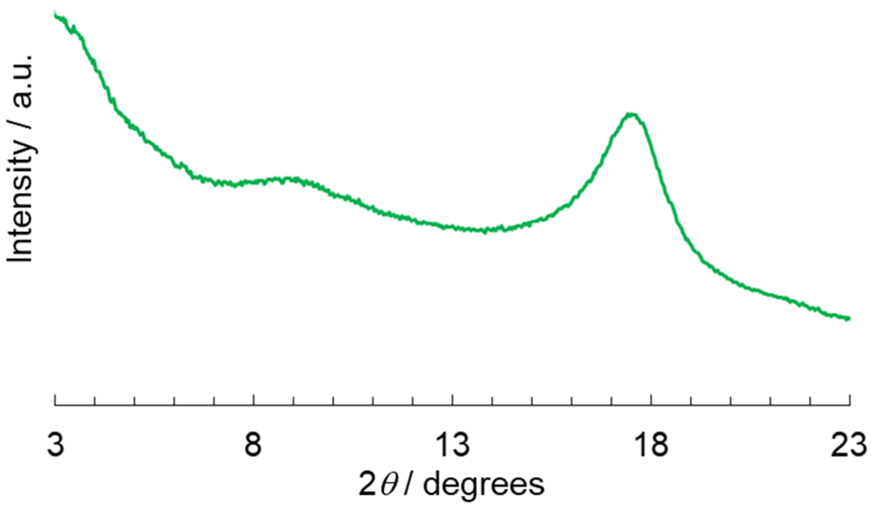

Fig. S4. X-ray diffraction pattern of C-PdDI (Converted from 2D scattering image).

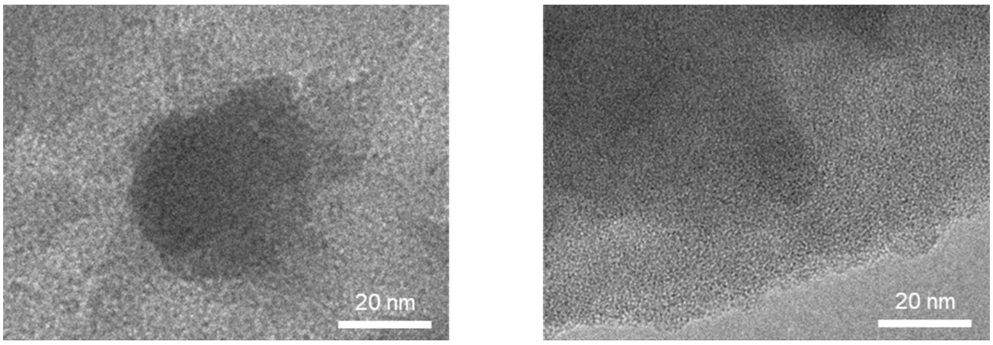

Fig. S5. High-resolution TEM images of C-PdDI.

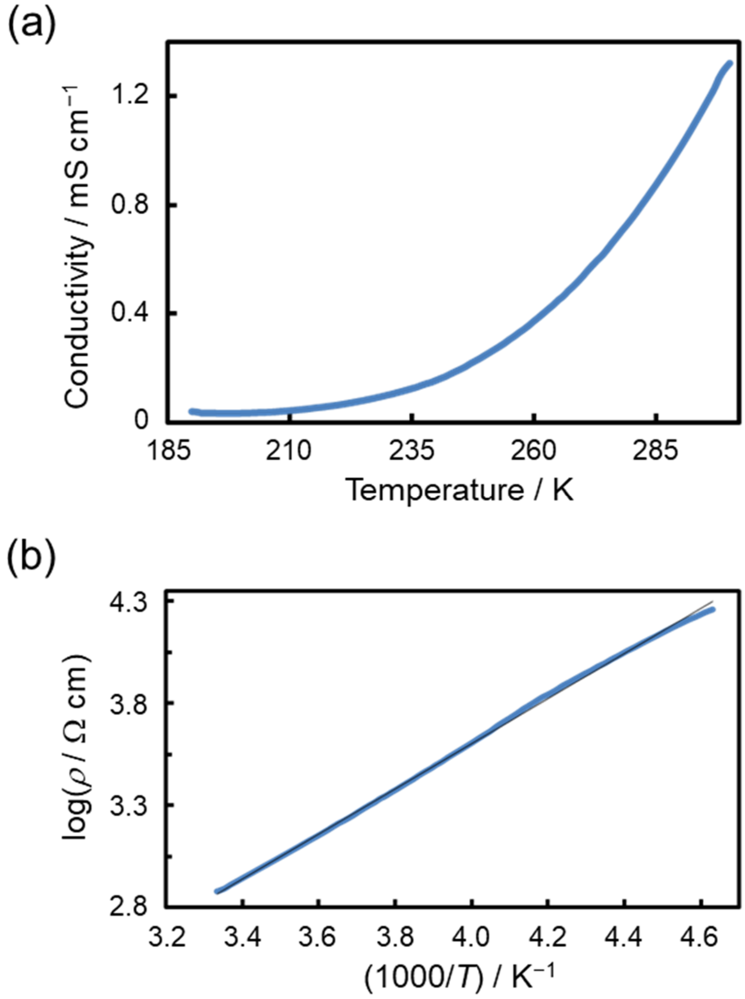

Fig. S6: Temperature-dependent conductivity of pelletized C-PdDI nanosheet measured using a four-probe method under helium. The electrical conductivity of the C-PdDI increased with temperature and was 1.3×10^{-3} S cm^{-1} at 300 K, indicating its semiconducting nature with the activation energy of 0.22eV. The activation energy was calculated as follows: Gradient of ln(Resistivity) graphs = $E_a/1000k_BT$; E_a = 0.22 eV.

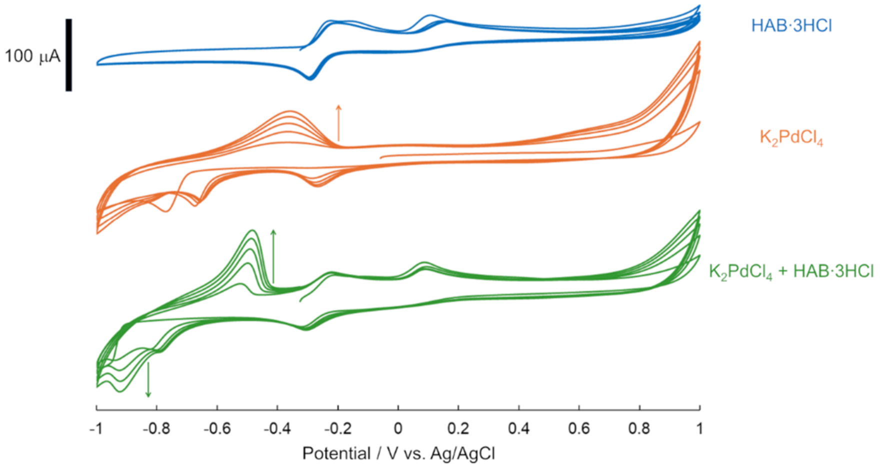

Fig. S7. Cyclic voltammograms of HAB·3HCl (blue line) and K_2PdCl_4 (orange line) dissolved in 0.1M $NaBF_4$/0.1 M NH_3 aqueous solutions and the precursor solution for preparing E-PdDI (K_2PdCl_4 + HAB·3HCl, green line).

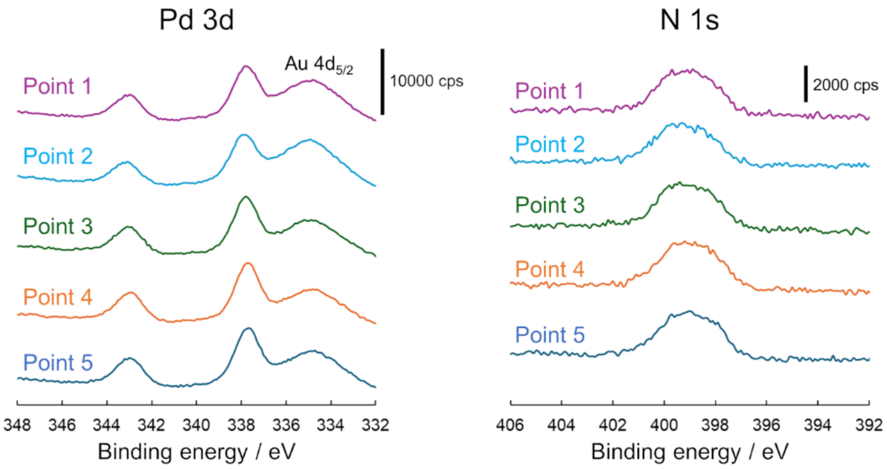

Fig. S8. X-ray photoelectron spectra of E-PdDI on an Au/glass electrode recorded at multiple points.

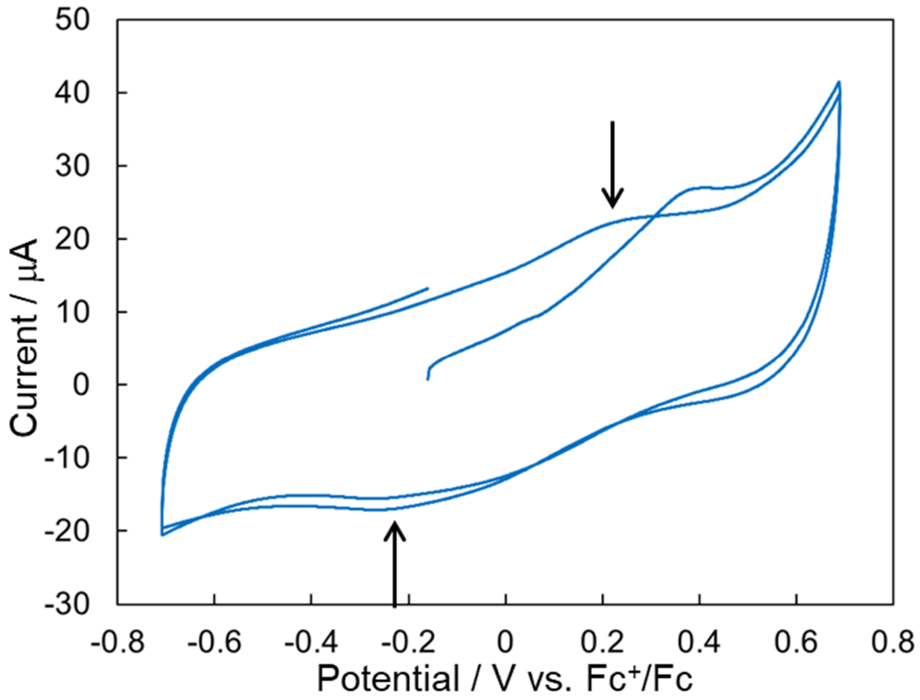

Fig. S9. Cyclic voltammograms of E-PdDI on an Au/glass in 1 M $^{n}Bu_4NPF_6/CH_3CN$.

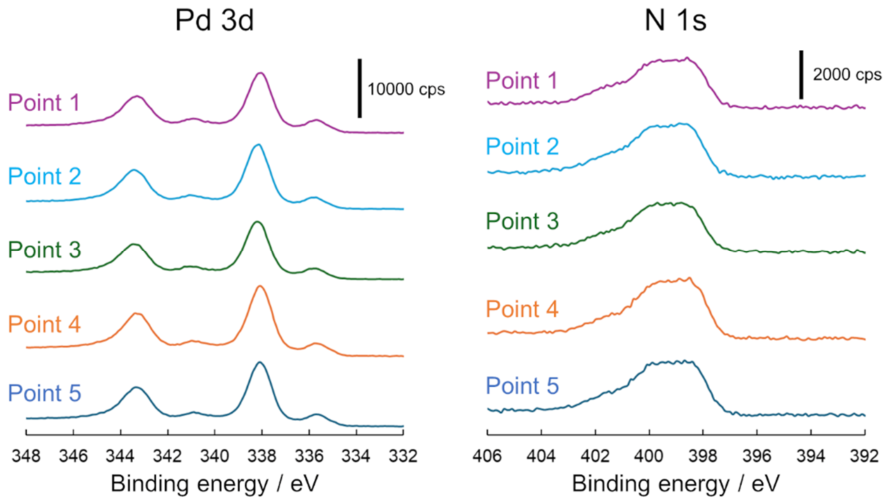

Fig. S10. X-ray photoelectron spectra of E-PdDI on a GC RDE after the cyclic voltammetry treatment recorded at multiple points.

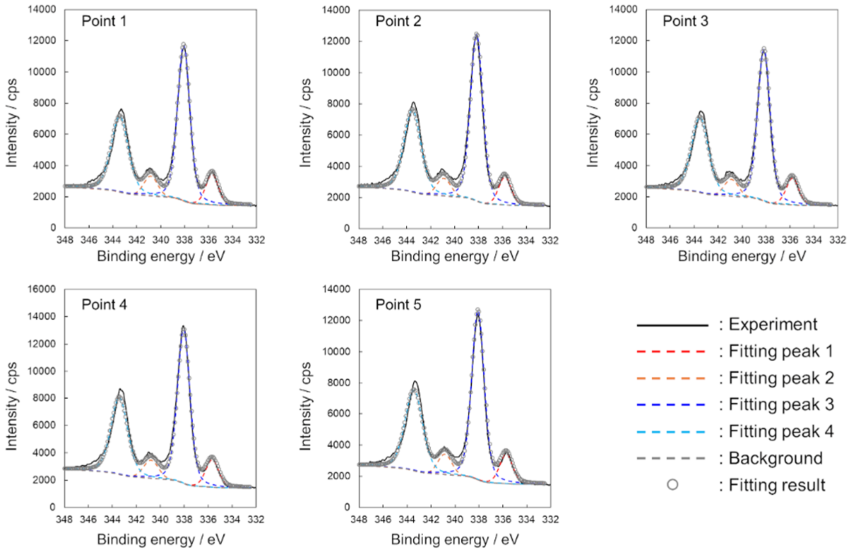

	Peak area				Pd (0) ratio / %
	Peak 1 (S_1)	Peak 2 (S_2)	Peak 3 (S_3)	Peak 4 (S_4)	$(S_1+S_2)/(S_1+S_2+S_3+S_4) \times 100$
Point 1	2765	1853	15026	10028	15.6
Point 2	2466	1652	16082	10775	13.3
Point 3	2429	1627	14732	9870	14.2
Point 4	3050	2044	17045	11420	15.2
Point 5	2805	1879	16386	10979	14.6
Average					14.6 ± 0.9

Fig. S11. Peak fitting results of Pd 3d spectra shown in Fig. S10 and the estimation of Pd(0) ratio in E-PdDI on a GC RDE after the cyclic voltammetry treatment.

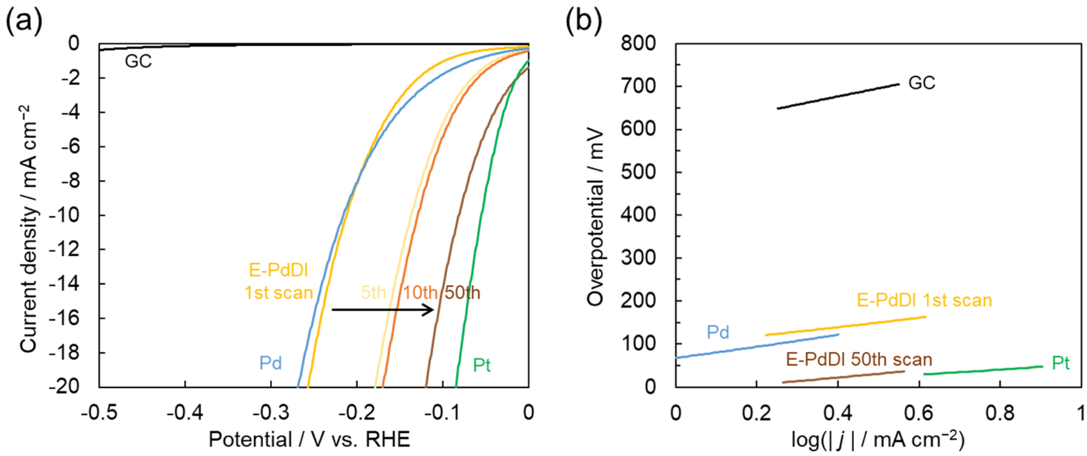

Fig. S12. (a) Linear sweep voltammograms recorded in a 0.5 M H_2SO_4 solution (pH = 0.48) at a rotating rate of 1600 rpm and (b) the Tafel plots of E-PdDI, GC, Pd, and Pt electrodes.

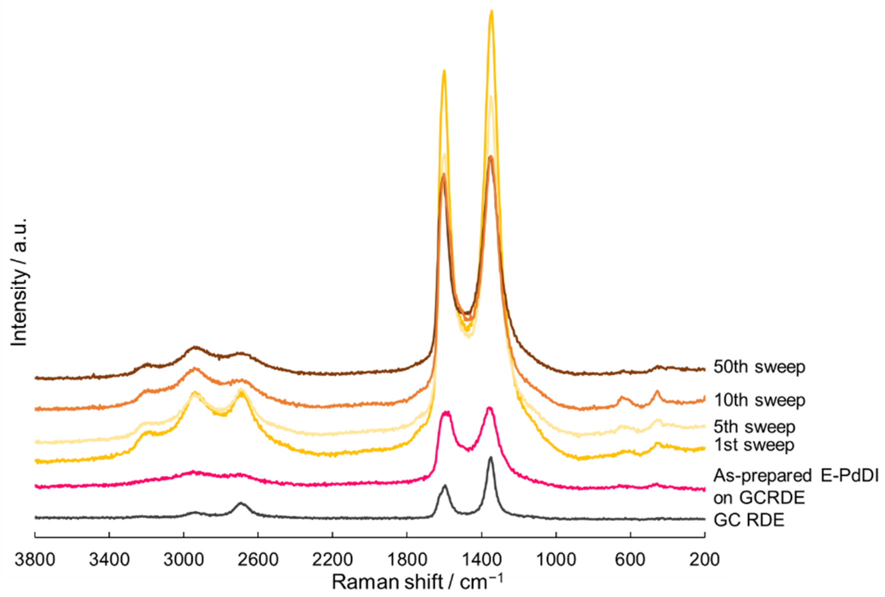

Fig. S13. Raman spectra after the 1st, 5, 10, and 50th potential sweep in the negative direction.

Table S1. Dependencies of the atomic ratios of Pd and N and the η_{10} on the number of potential sweeps for the activation process. The atomic ratios were calculated from Pd 3d and N 1s peaks in XPS.

Number of potential sweeps	0 (As-prepared)	1	5	10	50
Pd(0) ratio	0	0.20	0.77	0.79	1.55
Pd(II) ratio	1	1	1	1	1
N ratio	4.58	4.16	4.09	5.01	4.45
$Pd(0)_{atom\%}$	0	16.7	43.5	44.1	60.8
$\Delta Pd(0)_{atom\%}$	-	0	26.8	27.4	44.1
η_{10} / mV	-	215	139	131	84
$\lvert\Delta\eta_{10}\rvert$	-	0	76	84	131

$\Delta Pd(0)_{atom\%}$ and $|\Delta\eta_{10}|$ are defined as follows.

$\Delta Pd(0)_{atom\%}$ = ($Pd(0)_{atom\%}$ of E-PdDI after the *n*th sweep) − ($Pd(0)_{atom\%}$ of E-PdDI after the 1st sweep)

$Pd(0)_{atom\%}$ = Pd(0) ratio/(Pd(0) ratio + Pd(II) ratio)

$|\Delta\eta_{10}|$ = |(η_{10} of E-PdDI at the *n*th sweep) − (η_{10} of E-PdDI at the 1st sweep)|

(n = 5, 10, 50)

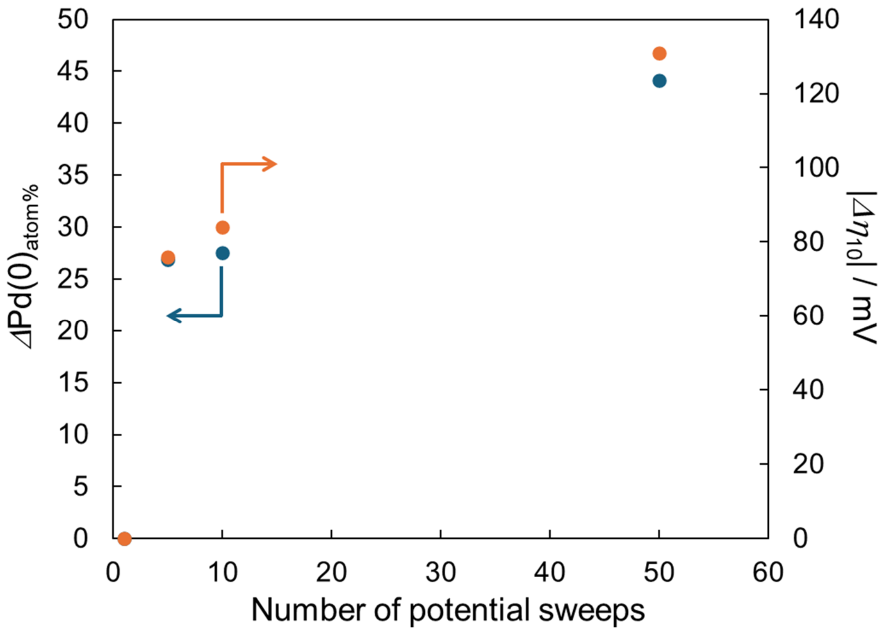

Fig. S14. Dependencies of $\Delta Pd(0)_{atom\%}$ (dark blue dots) and $|\Delta\eta_{10}|$ (orange dots) on the number of potential sweeps.

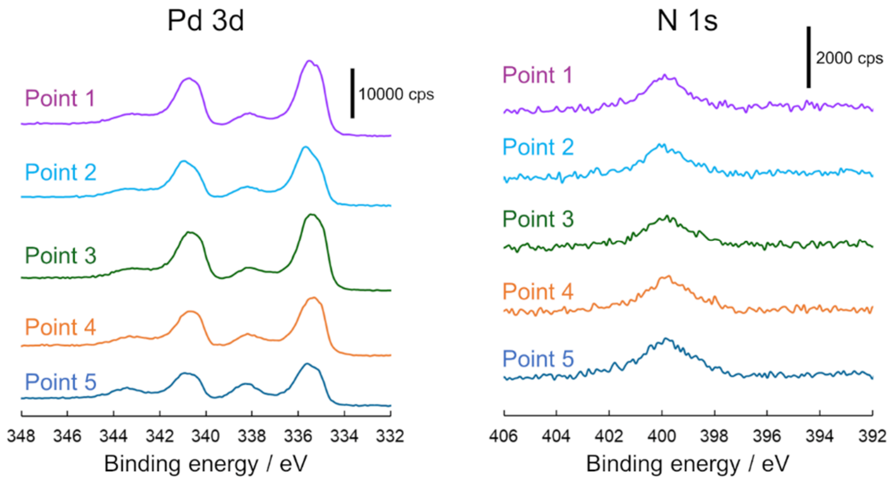

Fig. S15. X-ray photoelectron spectra of E-PdDI on a GC RDE recorded at multiple points after the 50th potential sweep.

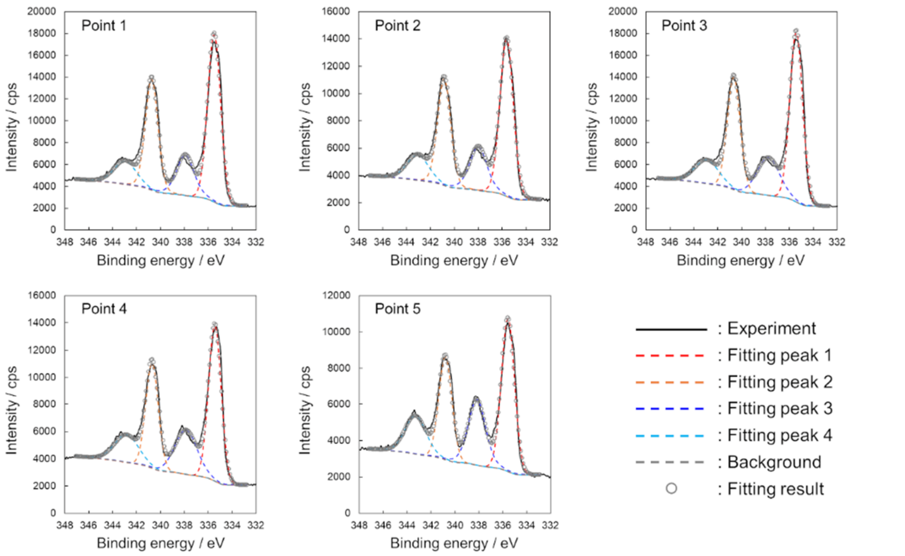

	Peak area				Pd (0) ratio / %
	Peak 1 (S_1)	Peak 2 (S_2)	Peak 3 (S_3)	Peak 4 (S_4)	$(S_1+S_2)/(S_1+S_2+S_3+S_4) \times 100$
Point 1	21128	14156	7713	5168	73.3
Point 2	16808	11262	6591	4416	71.8
Point 3	20918	14015	8524	5711	71.0
Point 4	15284	10240	7979	5346	65.7
Point 5	11694	7835	7198	4822	61.9
Average					69 ± 5

Fig. S16. Peak fitting results of Pd 3d spectra shown in Fig. S15 and the estimation of Pd(0) ratio in E-PdDI on a GC RDE after the 50th potential sweep.

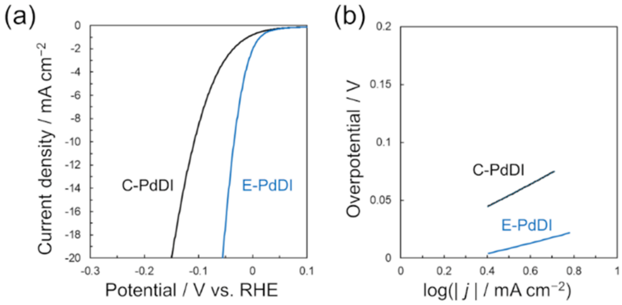

Fig. S17. (a) Linear sweep voltammograms and (b) the corresponding Tafel plots of C-PdDI and E-PdDI in a 0.5 M H_2SO_4 solution at a rotating rate of 1600 rpm.

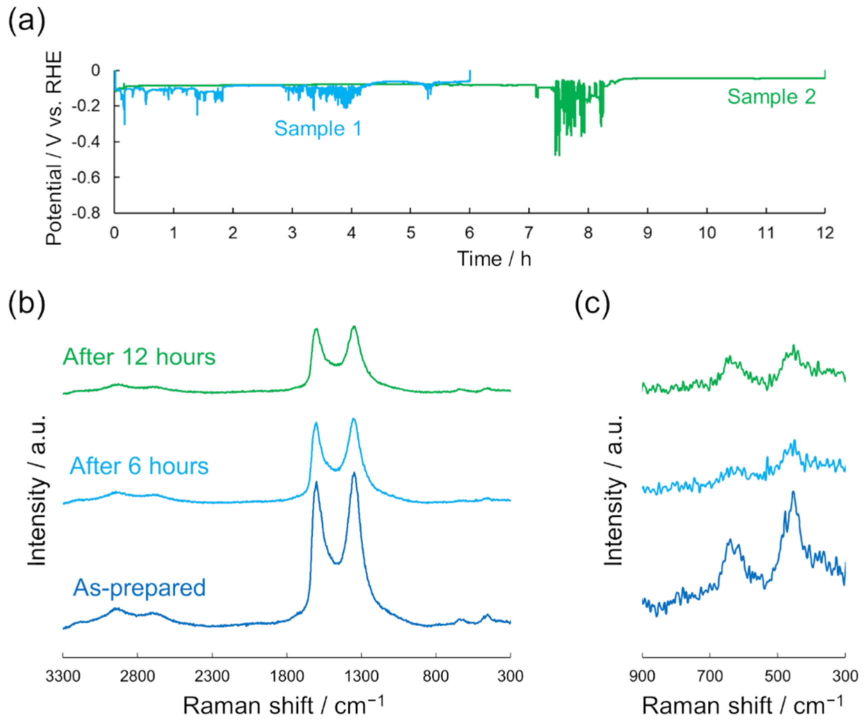

Fig. S18. (a) Chronopotentiograms of E-PdDIs (samples 1 and 2) recorded at the $j = -10$ mA cm^{-2} at a rotational rate of 1600 rpm in 0.5 M H_2SO_4 solution. (b) Raman spectra of as-prepared E-PdDI and E-PdDIs after 6- and 12-hour chronopotentiometry measurements. (c) Magnified Raman spectra in the region between 300 and 900 cm^{-1}.

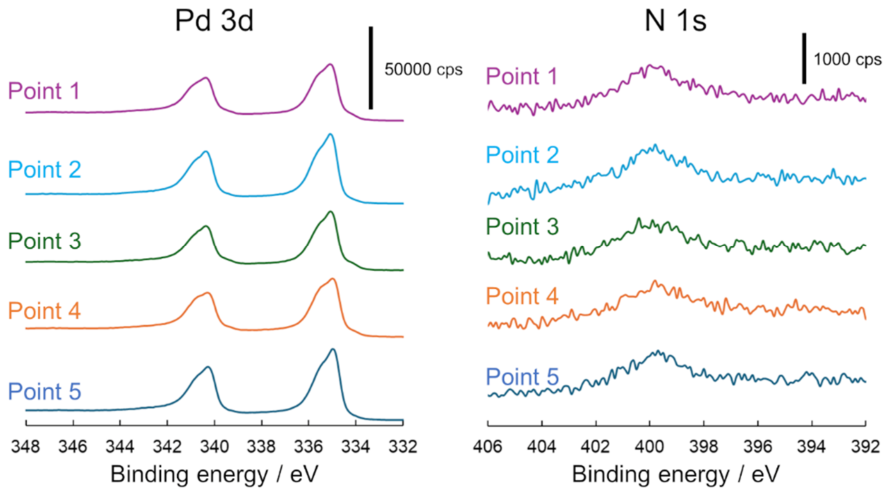

Fig. S19. X-ray photoelectron spectroscopy of E-PdDI on a GC RDE after a 12-hour chronopotentiometry measurement recorded at multiple points.

Calculation of the number of Pd atoms in the unit area from the ICP measurements

As described in the experimental section in the main text, the number of Pd atoms in the unit area (N_{pd} atom cm^{-2}) was calculated from the following equation:

$$N_{Pd} = \frac{c \times 10^{-6} \times 50 \times N_A}{M_{Pd} \times 1000 \times (0.2 \times 0.2 \times \pi)}$$

Where c, N_A, and M_{Pd} indicate the concentration of Pd detected by ICP measurement (ppb), the Avogadro constant (6.02×10^{23} atom mol^{-1}), and the molar mass of Pd (106.42 g mol^{-1}). The calculated N_{Pd} values are summarized in Table S2.

Table S2. N_{Pd} values for E-PdDIs after the CV treatment, 50th potential sweep, and 12-hour chronopotentiometry measurement.

	c / ppb	N_{Pd} / atom cm^{-2}
E-PdDI after CV treatment	80 ± 4	1.8×10^{17}
E-PdDI after 50th potential sweep	80 ± 30	1.8×10^{17}
E-PdDI after 12-hour chronopotentiometry	73 ± 1	1.6×10^{17}

Turnover frequency (TOF) calculation

TOF can be calculated by the following equation (1).[53]

$$TOF = \frac{j}{nFN} \quad (1)$$

Where j is the current density, n is the stoichiometric number of electrons (n = 2 for the HER), F is the Faraday constant (96500 C mol^{-1}), and N is the number of active sites in a unit area (mol cm^{-2}). The number of active sites of E-PdDI and Pd electrode were estimated as follows.

<E-PdDI>

According to the ICP measurements, the number of Pd atoms of E-PdDI-modified GC RDEs after CV treatment is 1.8×10^{17} atom cm^{-2} (Table S2). Multiple-point XPS analysis of E-PdDI after the 50th potential sweep exhibited that ca. 69 % of the Pd atoms took Pd(0) species, which are considered to perform as active sites (Figures S15 and S16). Hence, the TOF of E-PdDI can be calculated as follows (2).

$$TOF = \frac{j}{nFN} = \frac{10 \times 10^{-3} (A/cm^2)}{2 \times 96500 \times \left(\frac{1.8 \times 10^{17} \times 0.69}{6.02 \times 10^{23}}\right)} = 0.25\ (s^{-1}) \quad (2)$$

<Pd electrode>

The Unit cell parameter of Pd is a = 3.89 Å with the Fm−3m space group. We consider that only Pd atoms on the topmost surface in the Pd(111) plane contribute to the HER catalytic activity.

Two Pd atoms are present in the unit cell on the Pd(111) plane. Hence, the total number of Pd atoms in the unit area is calculated as follows (3).

$$\frac{2\ (atom/unit) \times 10^{-4}\ (m^2)}{\frac{(3.89 \times 10^{-10} (m) \times \sqrt{2})^2 \times \sqrt{3}}{4}} = 1.5 \times 10^{15}\ (atom\ cm^{-2}) \quad (3)$$

At −34 mV where E-PdDI exhibits j = 10 mA cm^{-2}, the Pd electrode showed j = 0.52 mA cm^{-2}. Hence, the TOF value of the Pd electrode at this potential is calculated as follows (4).

$$TOF = \frac{j}{nFN} = \frac{0.52 \times 10^{-3} (A/cm^2)}{2 \times 96500 \times \left(\frac{1.5 \times 10^{15}}{6.02 \times 10^{23}}\right)} = 1.1\ (s^{-1}) \quad (4)$$

[53] S. Shin, Z. Jin, D. H. Kwon, R. Bose, Y.-S. Min, *Langmuir* **2015**, *31*, 1196.
